# Accumulation of a Bioactive Benzoisochromanequinone Compound Kalafungin by a Wild Type Antitumor-Medermycin-Producing Streptomycete Strain

**DOI:** 10.1371/journal.pone.0117690

**Published:** 2015-02-19

**Authors:** Jin Lü, Qiang He, Luyao Huang, Xiaofeng Cai, Wenwen Guo, Jing He, Lili Zhang, Aiying Li

**Affiliations:** 1 State Key Laboratory for Microbial Technology, Shandong University-Helmholtz Joint Institute of Biotechnology and School of Life Sciences, Shandong University, Jinan 250100, China; 2 School of Life Sciences, Central China Normal University, Wuhan 430079, China; 3 State Key Laboratory of Agricultural Microbiology, Huazhong Agricultural University, Wuhan 430070, China; 4 Xinjiang Production & Construction Corps Key Laboratory of Protection and Utilization of Biological Resources in Tarim Basin, Tarim University, Alar 843300, Xinjiang, China; University of New South Wales, AUSTRALIA

## Abstract

Medermycin and kalafungin, two antibacterial and antitumor antibiotics isolated from different streptomycetes, share an identical polyketide skeleton core. The present study reported the discovery of kalafungin in a medermycin-producing streptomycete strain for the first time. A mutant strain obtained through UV mutagenesis showed a 3-fold increase in the production of this antibiotic, compared to the wild type strain. Heterologous expression experiments suggested that its production was severely controlled by the gene cluster for medermycin biosynthesis. In all, these findings suggested that kalafungin and medermycin could be accumulated by the same streptomycete and share their biosynthetic pathway to some extent in this strain.

## Introduction

Streptomycetes are a large group of Gram positive filamentous bacteria dwelling widely in soils, sediments and even some extreme environments. The structurally and functionally diverse natural products discovered in these microbes have been the major source of drug leads in the last decades for their antimicrobial, pesticidal, antineoplastic, anti-inflammatory, antivirus activity and so on [1–2].

It is noteworthy that some structurally-identical natural products could be isolated from different streptomycete strains, as well as many streptomycete species could synthesize more than one bioactive product [3]. Genome sequencing indicated the existence of a large number of cryptic gene clusters in *Streptomyces*, which opened up a new hotspot of genome mining in drug discovery in recent years [1, 4–5].

Aromatic polyketide antibiotics produced by *Streptomyces* are a structurally and functionally diverse class of secondary metabolites. Their polyaromatic carbon skeletons are biosynthesized by polyketide synthases (PKS), ketoreductases, aromatases and cyclases via a serial of earlier biosynthetic reactions. Subsequently, these carbon skeletons further undergo structural modifications in tailoring steps including oxygenation, dimerization, glycosylation, methylation and so on. These tailoring modifications resulted in a high diversity in the structure and biological activity of aromatic polyketide antibiotics [6].

**Fig 1:**
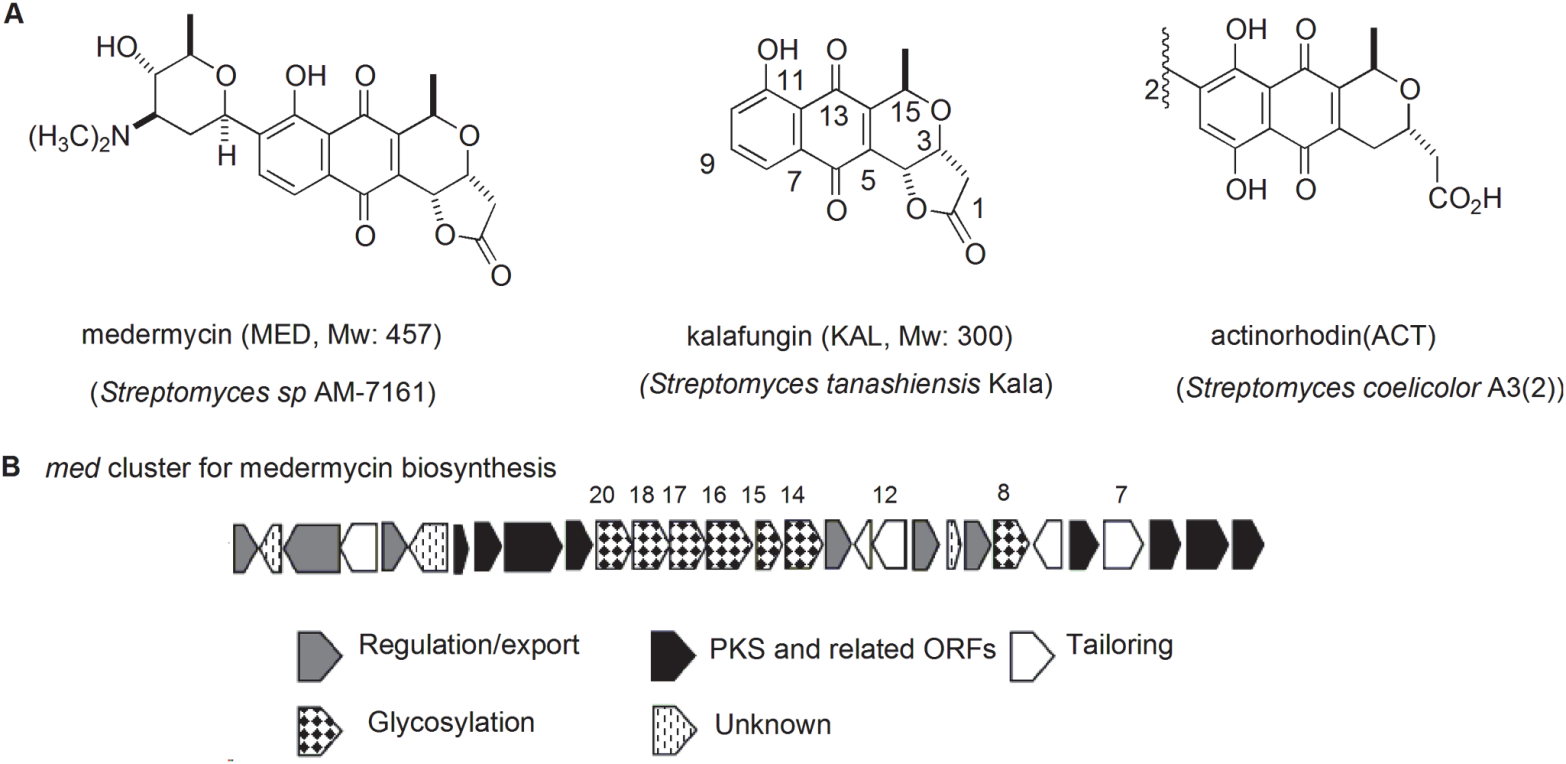
Structures of several important BIQ antibiotics and medermycin biosynthetic gene cluster. (A) Structures of three BIQ antibiotics and their producing strains. The numbering given is based on the biosynthetic origin of these compounds. (B) Organization of the gene cluster for medermycin biosynthesis. The genes (*med*-ORF8, 14, 15, 16, 17, 18 and 20) indicated with numbers are involved in *C*-glycosylation and *med*-ORF7 for oxygenation in the tailoring steps in the biosynthesis of medemycin.

Benzoisochromanequinones (BIQs, also referenced as pyranonaphthoquinones), generated by *Streptomyces*, represent a class of aromatic polyketide antibiotics [7–8]. This family includes medermycin (MED), kalafungin, granaticin, actinorhodin (ACT) and so on (Fig. 1A). Medermycin, a *C*-glycosylated antibiotic, shares an identical polyketide skeleton core with kalafungin. Actinorhodin is a well-known compound as a model for studying on biosynthetic mechanisms of aromatic polyketide antibiotics. All BIQ members possess strong antibacterial activity and have a distinct fused three-ring structure composed of a benzene, a quinone and a stereospecific pyran ring (Fig. 1A).

Medermycin and kalafungin were found to show significant antitumor activity and were able to inhibit the proliferation, invasion and metastasis of many types of tumor cells through a novel alkylation mechanism [9–10]. They were isolated originally from different streptomycete species, respectively [11–12], and have not been reported to accumulate in a same strain.

In 2003, a 29-gene-containing entire medermycin biosynthetic gene cluster (*med* cluster) was cloned from a medermycin-producing strain *Streptomyces sp*. AM-7161 (AM-7161) [13] (Fig. 1B), whilst the cloning and sequencing of the complete kalafungin gene cluster has not been reported, except that only a 14 kb DNA fragment involved in the biosynthesis of kalafungin was cloned from a kalafungin-producing strain *Streptomyces tanashiensis* Kala DSM731 (DSM731) [14–15].

In the course to investigate the biosynthetic mechanism of medermycin [16–18], we found that medermycin titre was quite low in the wild type strain AM-7161. Meanwhile, an unknown product (X) was always accumulated in this strain with an even higher yield than medermycin. Applying heterologous expression, UV mutagenesis and NMR spectrometry, we revealed that AM-7161 could produce at least two antibiotics medermycin and kalafungin which share partially the same biosynthetic pathway.

## Materials and Methods

### Strains, plasmids and reagents

All bacteria strains are listed in table 1: *Streptomyces sp*. AM-7161 (AM-7161) and *Streptomyces tanashiensis* Kala DSM731 (DSM731) were wild type strains able to produce medermycin and kalafungin respectively [11–13]; *Streptomyces coelicolor* CH999 (CH999) and *Streptomyces lividans* K4–114 (K4–114) were hosts for heterologous expression of *med* cluster [19]; *Staphylococcus epidermidis* ATCC 35984 was used for antibacterial activity test [20]; *Escherichia coli* ET12567/pUZ8002 was used for intergeneric conjugation [19]; pIK340 is a plasmid carrying an entire medermycin biosynthetic gene cluster [13].

**Table 1:** Bacteria used in this study.

Strains	Property descriptions	References
*Streptomyces* sp. AM-7161	A wide type medermycin-producing strain	[11,13]
*Streptomyces tanashiensis* Kala DSM731	A wide type kalafungin-producing strain	[12]
*Streptomyces coelicolor* CH999	An actinorhodin-gene-cluster-deficient mutant used as a common host for the biosynthesis of polyketide compounds	[19]
*Streptomyces lividans* K4–114	A host commonly used for heterologous expression due to its efficient genetic manipulation system	[19]
*Streptomyces sp* AM-7161-M1	A mutant derived from *Streptomyces* sp. AM-7161, through mutagenesis	This study
*Streptomyces sp* AM-7161-M2	A mutant derived from *Streptomyces* sp. AM-7161, through mutagenesis	This study
*Streptomyces sp* AM-7161-M3	A mutant derived from *Streptomyces* sp. AM-7161, through mutagenesis	This study
*Staphylococcus epidermidis* ATCC 35984	An indicator strain used in plate assays of antibacterial activity of mutants from AM-7161	[20]
*Escherichia coli* ET12567/pUZ8002	A strain commonly used for conjugation between *E. coli* and *streptomyces*	[19]

### Cultivation of bacteria

Media for streptomycete cultivation include YEME, SFM, GYM, SEED medium and R4 medium [18–19, 21]. *E. coli* and *S. epidermidis* ATCC 35984 were cultivated in LA agar or liquid medium [22].

*Streptomyces* spores were inoculated on solid or in liquid media and grown at 30°C while *E. coli* and *S. epidermidis* ATCC 35984 were grown at 37°C [19, 22]. When needed, media were supplemented with antibiotics at a working concentration of 25 μg mL^-1^ for kanamycin, 25 μg mL^-1^ for chloramphenicol, 100 μg mL^-1^ for nalidixic acid and 100 μg mL^-1^ for ampicillin respectively.

### Genetic manipulations

General genetic manipulations in *E. coli*, including DNA isolation, enzyme digestion/ligation and DNA transformation, were conducted according to previous descriptions [22].

Genomic DNAs were isolated from *Streptomyces* using 2 mg mL^-1^ lysozyme to remove the cell wall [19]. Amplification of *Streptomyces* genes with high (G+C) % by PCR was performed using KOD-Plus polymerase (Toyobo). Protoplast transformation and intergeneric conjugation for introduction of DNA into *Streptomyces* hosts were carried out according to standard protocols [19].

### Heterologous expression of the entire gene cluster for medermycin biosynthesis

pIK340 was introduced into *E. coli* ET12567/pUZ8002, then transferred into CH999 and K4–114 by intergeneric conjugation, respectively [19]. The proposed conjugants were confirmed through kanamycin-resistant selection and PCR amplification. The recombinant strains CH999/pIK340 and K4–114/pIK340 were cultivated on R4 solid medium for morphological observation of a brown pigment indicating medermycin production [13], and cultivated in R4 liquid medium for metabolite analysis.

### Metabolite analysis of streptomycete cultures

50 μL of streptomycete spores (10^6^ spores μL^-1^) were inoculated into 5 mL of SEED medium and incubated for 2 d on a shaker (220 rpm) at 30-, then transferred into 45 mL of R4 liquid medium. After 5 d cultivation at 30-, streptomycete cultures were centrifuged at 6 000 rpm for 10 min. LC/APCI/MS analysis of the supernatants using Agilent 1100 HPLC/Brucker Esquire HCT was performed under the following conditions: column TSK-Gel 100 ODS (5 μm, 4.6 mm I.D.×15 cm), solvents (A: H_2_O containing 0.5% glacial acetic acid, B: CH_3_CN containing 0.5% glacial acetic acid), gradient profiles (0–5 min, 20% B; 5–25 min 20–70% B; 25–28 min 70–95% B; 28–32 min 95% B; 32–35 min 95–20% B; 35–40 min 20% B), column temperature: 40-, flowing rate: 1 mL min^-1^, and monitor: 254 nm and 430 nm.

In order to compare the yield of the target compound between the wild type strain and mutant strain by HPLC analysis, the values of Sm/Sw (Sm: peak area for the mutant strain; Sw: peak area for the wild type strain) were calculated. The experiments were performed in triplicate.

### UV mutagenesis of streptomycete spores

5 mL of streptomycete spore samples (10^5^ spores μL^-1^) of AM-7161 loaded in a Petri dish (5 cm in diameter) were radiated for 0 s, 20 s, 40 s, 60 s and 80 s respectively using a UV light (253.7 nm) at a distance of 30 cm between UV light and samples. Then, spore samples were diluted and spread onto GYM agar plates for 2 d cultivation in dark at 28-. Under radiation conditions causing a mortality rate of 96.9% (wavelength: 253.7 nm, radiation distance: 30 cm, radiation duration: 40 s), mutagenized spores were diluted and cultivated on R4 agar medium. Three mutant colonies showing significant change of pigmentation were selected for antibacterial activity test and metabolite analysis.

### Plate assay of antibacterial activity

After 4 d cultivation in R4 liquid medium, the supernatants of streptomycete cultures were extracted with EtoAc and re-dissolved in 1 mL of EtoAc. Meanwhile, several 0.6-cm-in-diameter sterile filter papers were placed onto the surface of LA agar medium, on which *S. epidermidis* ATCC 35984 cells were spread already. Subsequently, 5 μL of crude extracts (ca. 8 mg mL^-1^) from streptomycete cultures were spotted on the center of each filter paper, as well 5 μL of EtoAc was used as blank control. Then, the agar plates were incubated at 37°C until confluent bacterial growth was observed and inhibition zones around the filter papers appeared. The values of Dm/Dw (Diameter of inhibition zone by the mutant strain/Diameter of inhibition zone by the wild type strain) were calculated. The experiments were performed in triplicate.

### Purification of compound X

Freshly-prepared spores of AM-7161 were inoculated on totally 40 L of R4 solid medium (40 mL of medium for each Petri dish) and incubated for 5 d at 30°C. Then, solid cultures were cut into small pieces and soaked for 6 h in EtoAc at room temperature, followed by 30 min sonication. Finally, 120 L of EtoAc crude extracts were collected after extracting for three times and evaporated. Subsequently, 6.15 g dried sample was subjected to macroporus resin chromatography (MCI-GEL CHP20/P120, Mitsubishi) through a gradient elution with methanol and water. All the fractions showing antibacterial activity measured as descriptions in the section 2.7 were collected respectively and evaporated, followed by further fractionation through silica gel column chromatography (Qingdao Haiyang Chemicals & Silica gel Co. Ltd, China) with a step elution gradient using CHCl_3_ and methanol (1000:1–100:1).

After the fractions showing antibacterial activity were collected and re-dissolved in methanol containing 10% DMSO, they were subjected to Sephadex LH-20 chromatography (GE Healthcare Bio-Sciences AB, Sweden) and eluted with 100% methanol to collect the fraction of compound X.

### HPLC/HRMS (High Resolution Mass Spectrum) analysis of compound X

HPLC/HRMS measurement was conducted on the Ailent 6530 Accurate-Mass Quadrupole Time-of-Flight (Q-TOF) equipped with Agilent 1260 HPLC in a full scan in positive mode (ESI+). During HPLC analysis, purified compound X was detected at 254 nm and eluted with a 40 min gradient as described as above (section: *Metabolite analysis of streptomycete cultures*) through a Shim-pack VP-ODS column (5 μm, 250 L×4.6 mm, Shimadzu). During subsequent HRMS analysis, the parent ion with m/z 301.0713 for compound X (C_16_H_13_O_6_ ([M+H]^+^)) at ca.21.8 min retention time was monitored.

### NMR spectroscopy

^1^H and ^13^C NMR spectra of compound X were recorded on Agilent Bruker AV500 instrument. Data for compound X: ^1^H NMR (500 MHz, CDCl_3_) δ 1.574 (3H, d, *J* 7.0 Hz, 15-CH_3_), 2.710 (1H, d, *J* 18.0 Hz, 2-*H*_a_H_b_), 2.985 (1H, dd, *J* 18.0 and 5.0 Hz, 2- H_a_ *H*_*b*_), 4.699 (1H, dd, *J* 5.0 and 3.0 Hz, 3-H), 5.095 (1H, q, *J* 7.0 Hz, 15-H), 5.269 (1H, d, *J* 3.0 Hz, 4-H), 7.306 (1H, dd, *J* 8.0 and 1.5 Hz, 10-H), 7.683 (1H, dd, *J* 8.50 and 7.50 Hz, 9-H), 7.701 (1H, dd, *J* 7.50 and 1.50 Hz, 8-H), 11.823 (1H, s, 11-OH); ^13^C NMR (125 MHz, CDCl_3_) δ 18.55, 36.87, 66.24, 66.45, 68.61, 114.81, 119.71, 124.84, 131.47, 135.14, 137.17, 149.73, 161.89, 173.91, 181.47 and 187.00. The spectroscopic data (^1^H and ^13^C NMR) are in close agreement with the literatures [8].

## Results

### Discovery of an unknown compound X in a medermycin-producing strain AM-7161

Firstly, the crude extract of AM-7161’s fermentation culture was analyzed by LC/MS to investigate whether it could accumulate any other natural products besides medermycin.

**Fig 2:**
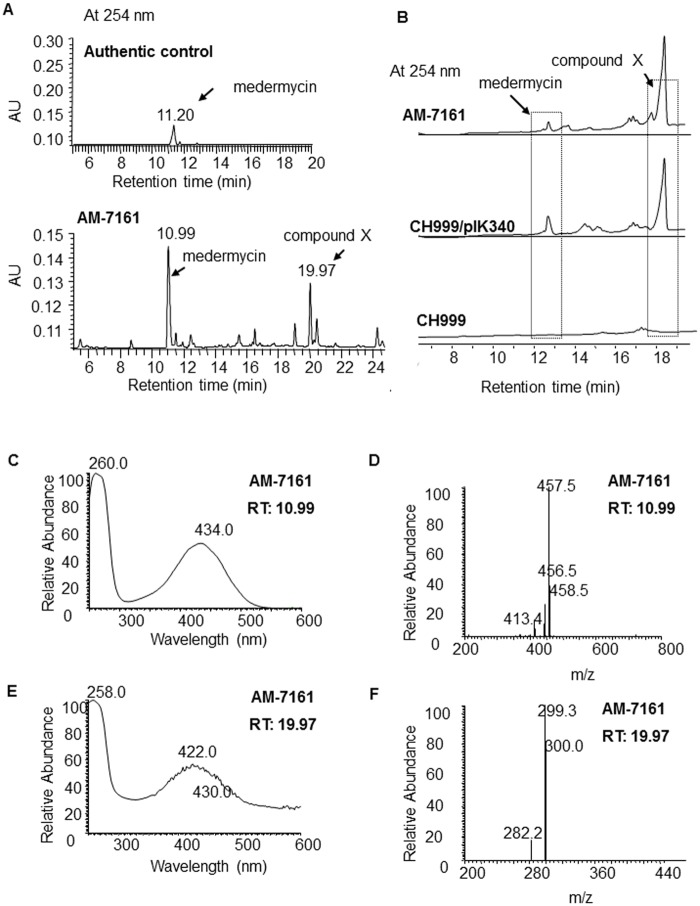
LC/MS analysis of the metabolites by the medermycin-producing *Streptomyces sp*. AM-7161 and heterologous expression strain. (A) UV absorption at 254 nm of authentic medermycin (upper) and the metabolites by *Streptomyces sp*. AM-7161 (bottom) (medermycin has a characteristic absorption at 254 nm). (B) UV absorption at 254 nm of metabolites by the wild type strain AM-7161 and heterologous expression strain CH999/pIK340. (C and E) Full-wavelength absorption spectra for two peaks in Fig. 2A at 10.99 min and 19.97 min respectively. (D and F) MS spectra of two peaks in Fig. 2A at 10.99 min and 19.97 min respectively.

Fig. 2A showed that AM-7161 could produce medermycin as expected, indicated by the peak at 10.99 min, which was verified by its full-wavelength absorption and mass spectra in Fig. 2C-D.

Fig. 2A also showed the production of an unknown compound (X) in AM-7161, indicated by an obvious peak at 19.97 min. Under different fermentation conditions, the production of medermycin and compound X in AM-7161 showed different yields: Given higher production of medermycin, compound X showed a lower yield (Fig. 2A, bottom for AM-7161). In contrast, its production was very high as the yield of medermycin was lower (Fig. 2B, upper for AM-7161).

### Accumulation of compound X by heterologous expression of the medermycin biosynthetic gene cluster

Next, in order to clarify whether the biosynthetic pathways of compound X and medermycin were related, pIK340, an integrative plasmid harboring the entire *med* gene cluster, was delivered into two commonly-used streptomycete hosts, *S. coelicolor* CH999 and *S. lividans* K4–114.

As expected, the production of medermycin (indicated by the peak at 12.4 min in Fig. 2B) was detected in the recombinant strain CH999/pIK340. Besides medermycin, compound X was also accumulated by this recombinant strain and indicated by the peak at 18.4 min, in sharp contrast to the control CH999, which could not produce either medermycin or compound X (Fig. 2B). Another recombinant strain K4–114/pIK340 also gave a similar result (data not shown).

### Structural elucidation of compound X

**Fig 3:**
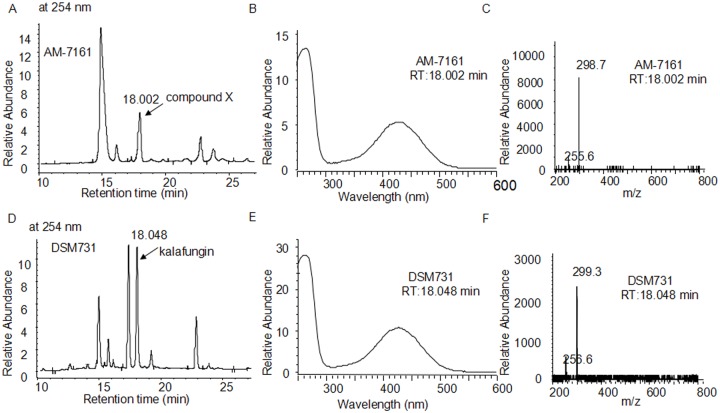
LC/MS analysis of the metabolites by the medermycin-producing *Streptomyces sp* AM-7161 (A, B and C) and kalafungin-producing *Streptomyces tanashiensis* Kala DSM731 (D, E and F). (A) and (D) UV absorption at 254 nm of metabolites by the wild type strain AM-7161 and DSM731. (B and E) Full-wavelength absorption spectra for two peaks in A at 18.002 min (compound X) and in D at 18.048 min (kalafungin) respectively. (C and F) MS spectra for compound X at 18.002 min in A and kalafungin at 18.048 min in D respectively.

Compound X produced by AM-7161 has a molecule weight (Mw) of 300 and characteristic absorption at 254 and 422 nm (Fig. 2E-2F). To identify compound X, we firstly used DSM731 as control and compared the properties between kalafungin by DSM731 and compound X by AM-7161 using LC/MS. Fig. 3A-3F showed that DSM731 gave a peak indicating kalafungin production with same retention time, absorption pattern and mass spectrum to compound X in AM-7161.

**Fig 4:**
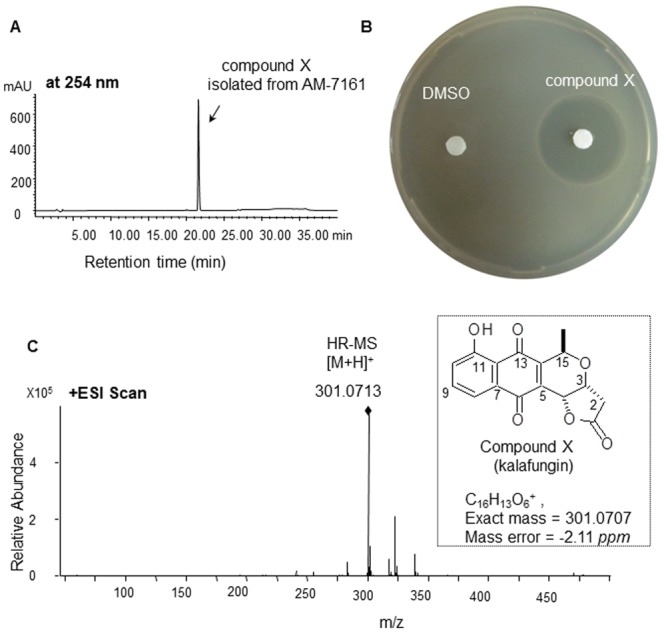
Purification and structural elucidation of compound X isolated from AM-7161. Absorption profile at 254 nm (A, HPLC analysis), bioassay (B, DMSO as blank control), and high resolution mass spectrum (C) of compound X purified from AM-7161 are shown.

To further identify the structure of compound X, we purified it from AM-7161 (Fig. 4A). Then we performed bioactivity test (Fig. 4B, using *S. epidermidis* ATCC 35984 as an indicator bacterium) and structural elucidation of purified compound X by both HRMS and NMR measurements. Fig. 4C showed the mass of compound X (found: 301.0713 and calculated: 301.0707 for C_16_H_13_O_6_ ([M+H]^+^)) from AM-7161 is quite close to that of kalafungin in the reference [8]. ^1^H and ^13^C NMR data (Fig. A and Fig. B in S1 File) further confirmed definitely its structure to be identical to that of kalafungin [8].

### UV mutagenesis and bioassay of streptomycetes

**Fig 5:**
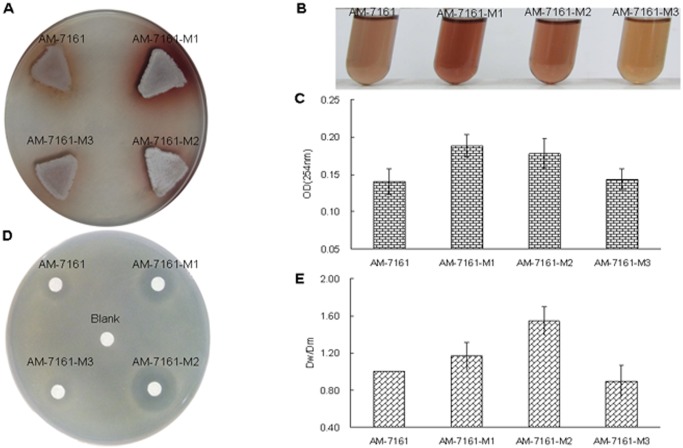
Comparison of pigmentation and bioassay between the wild type AM-7161 and mutant strains. (A) and (B) Pigmentation of AM-7161 and mutant strains on solid and liquid R4 medium. (C) Absorbance values at 254 nm of crude extracts from AM-7161 and mutant strains. (D) Plate assay of antimicrobial activity of AM-7161 and mutant strains against *S. epidermidis* ATCC 35984. EtoAc is used as blank control. (E) The ratio of the inhibition zone diameter by mutant strains (Dm) to that of the inhibition zone by AM-7161 (Dw).

Initially, in order to improve the production of medermycin, we conducted UV mutagenesis on AM-7161, and then obtained several isolates (AM-7161-M1, -M2 and-M3) which showed stronger pigmentation on R4 medium (Fig. 5A-B), indicating medermycin production [13].

We compared absorption values at 254 nm of liquid cultures of three mutants (Fig. 5C). Pigmentation and UV absorption for these strains suggested such a sequence: M1>M2>M3>AM-7161 (Fig. 5 A-C).

Next, we used *S. epidermidis* ATCC 35984 as an indicator bacterium for bioassay of these mutants. Comparison of Dm/Dw of inhibition zones between mutants and AM-7161 showed that antibacterial activity of these mutants was not in accordance with their pigmentation change: M2>M1>AM-7161>M3 (Fig. 5D-E).

### Metabolite analysis of three mutants using HPLC

**Fig 6:**
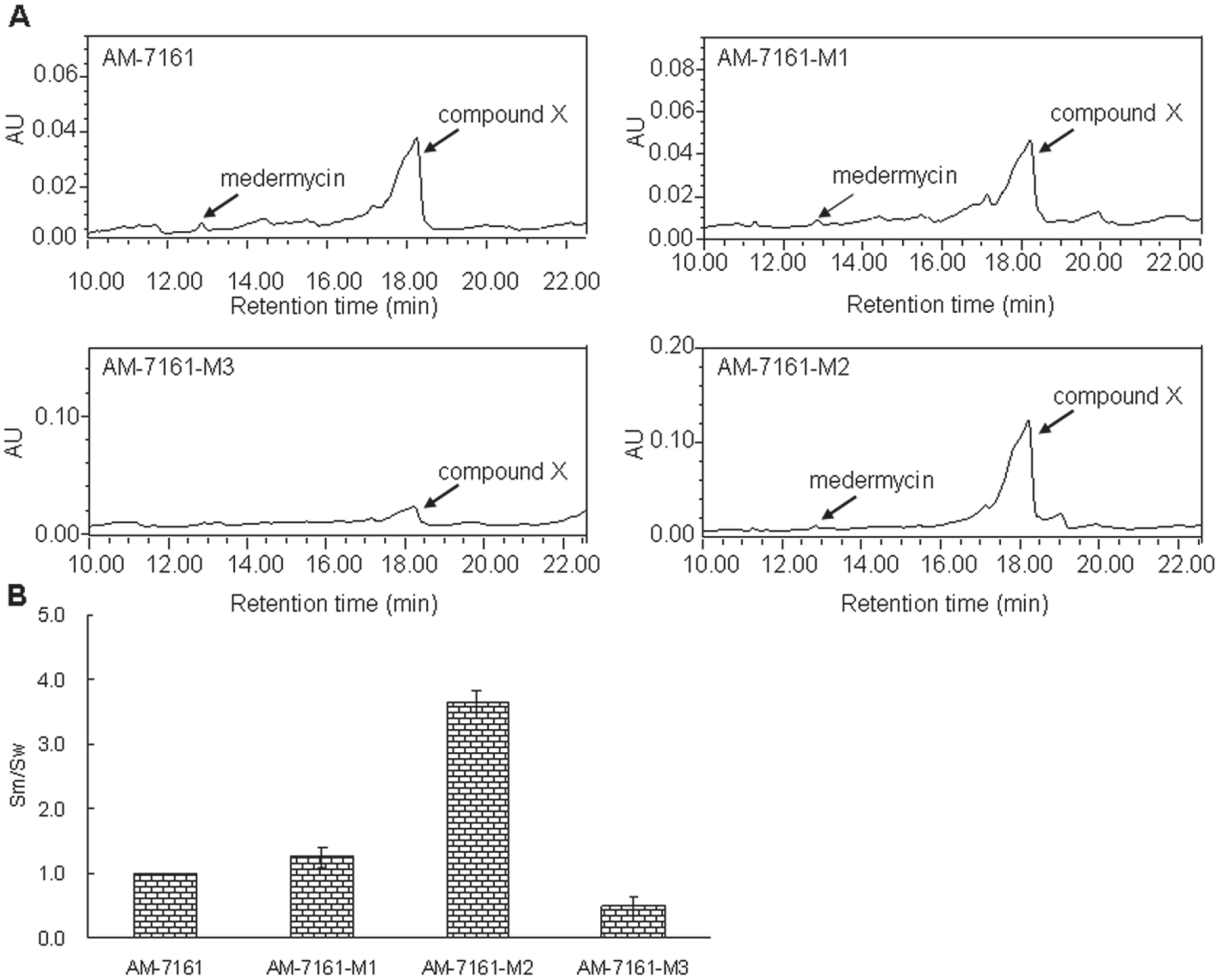
Metabolite analysis of AM-7161 and mutant strains by HPLC. (A) UV absorption at 254 nm of AM-7161 and mutant strains. (B) The ratio of the peak area (Sm) by mutant strains to the peak area (Sw) by AM-7161 at 18.2 min, indicating the production of compound X.

HPLC measurements were performed to analyze the yield change of medermycin and compound X in three mutants: Absorption profiles at 254 nm showed that the peak at 12.4 min indicating medermycin production had slight difference among these strains. On the contrary, the peak at 18.2 min representing compound X had significant change (Fig. 6A). According to the values of S_m_/S_w_ (S_m_: peak area at 18.2 min for mutant strains; S_w_: peak area at 18.2 min for the wild type strain AM-7161), M2 produced more compound X by 3 fold at least than AM-7161 (Fig. 6B).

## Discussion

Besides antifungal, antibacterial and antiprotozoa activity, kalafungin was reported to inhibit many types of tumor cells via a novel alkylation mechanism [9,10,12]. Because the value of IC_50_ of kalafungin against tumor cells is just slightly higher than that of medermycin and it has a more simple structure, it has been attracting more interests in pharmacological and toxicological studies, and even in the area of molecular drug designing [8–10].

### Accumulation of kalafungin in different actinomycetes

In the present study, we firstly elucidated that kalafungin could be produced by the medermycin-producing strain AM-7161, and we also obtained a kalafungin-high-yielding mutant strain through UV mutagenesis.

Though the production of kalafungin by AM-7161 has not been reported previously, several wild type actinomycete bacteria in nature were found to accumulate kalafungin, such as *Streptomyces tanashiensis* Kala DSM731 and *Streptomyces tanashiensis* NRRL B-1692 as well as *Nocardiopsis dassonvillei* subsp. prasina [24–25].

It is quite common that more than one bioactive compound could be accumulated in a same cell of *Streptomyces* species. Taken the type strain *S. coelicolor* as an example, besides four bioactive products including actinorhodin (Fig. 1B) isolated from it in a few decades ago, more natural products have been screened from it successfully, directed by genome mining approaches in recent years [23]. Hence, it was predictable that AM-7161 could accumulate more than one bioactive compound, as proved in the present work. Even we could not rule out the possibility that it might be able to produce more bioactive natural products.

### Production of kalafungin under the control of the medermycin gene cluster

Since medermycin and kalafungin possess an identical polyketide skeleton core, we suspected that they have a closer correlation in their biosynthetic pathways. But up to now, the cloning and DNA sequencing of the whole gene cluster for kalafungin biosynthesis have not been available. Hence, we still could not deduce its biosynthetic logic within the cell.

In order to find out the genetic origin of kalafungin in AM-7161, in the present study, we expressed the whole medermycin gene cluster cloned from AM-7161 in two commonly-used hosts and definitely demonstrated that the medermycin gene cluster makes essential and complete contributions to the production of kalafungin. Therefore, we could conclude that the production of kalafungin in AM-7161 was controlled by the medermycin gene cluster.

### Proposed biosynthetic routes of kalafungin and medemycin in AM-7161

**Fig 7:**
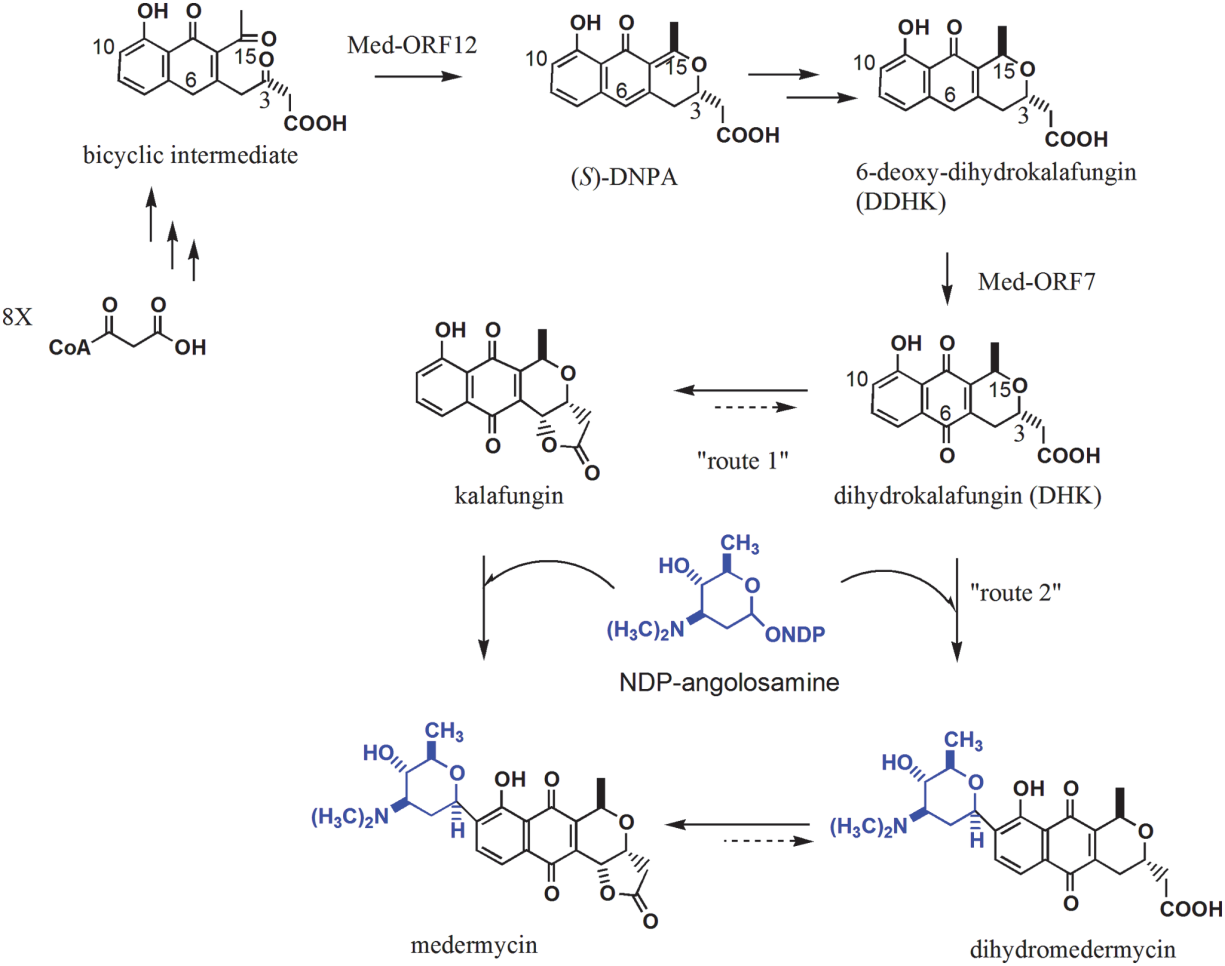
Proposed tailoring steps in the biosynthetic pathway of medermycin. (*S*)-DNPA: 4-dihydro-9-hydroxy-1-methyl-10-oxo-3-H-naphtho[2,3-c]pyran-3-acetic acid, as a first chiral intermediate in the pathway of medermycin. NDP-angolosamine: the specific glycosyl moiety formed under the control of seven glycosylating genes indicated in the *med* gene cluster in Fig. 1B. The conversion from DDHK to DHK could be catalyzed by an oxygenase encoded by *med*-ORF7 in the *med* gene cluster [26]. The numbering given is based on the biosynthetic origin of these intermediates.

Though the biosynthesis of medermycin has not been studied very deeply, we reported that the formation of a compound (*S*)-DNPA (4-dihydro-9-hydroxy-1-methyl-10-oxo-3-H-naphtho[2,3-c]pyran-3-acetic acid) could be catalyzed by a stereospecific ketoreductase Med-ORF12 encoded by the medermycin gene cluster, suggesting (*S*)-DNPA to be a first chiral intermediate in the biosynthetic pathway of medermycin, and also in the pathway of actinorhodin, a well-studied compound for its biosynthesis [18](Fig. 1B and Fig. 7).

After the *med* gene cluster was cloned and sequenced, dihydrokalafungin (DHK, a non-lactonized form of kalafungin) was proposed to be a further intermediate after (S)-DNPA and 6-deoxy-dihydrokalafungin (DDHK) (a non-lactonized and 6-deoxy form of kalafungin), and then to be *C*-glycosylated at C-10 in the biosynthetic pathway of medermycin (Fig. 7) [13].

In recent years, more and more evidences suggested that actinorhodin, medermycin and kalafungin should share common earlier biosynthetic stages from the carbon condensation till the formation of DDHK via (*S*)-DNPA (Fig. 7) [18, 26–27]. In 2013, Ichinose K et al. proved that an oxygenase Med-ORF7 encoded by the medermycin gene cluster could control subsequent oxygenation at C-6 of DDHK into DHK as an important intermediate [27] (Fig. 1B and Fig. 7).

Since medermycin was a glycosylated form of kalafungin which could be formed from DHK via spontaneous lactonization between C1 and C4 [7, 28], all evidences from previous studies [18, 26–28] and present experiments allowed us to deduce kalafungin to be either an intermediate (route 1) or shunt product (route 2) in the medermycin pathway in AM-7161 (Fig. 7): Route 1—-DHK was lactonized firstly to form kalafungin, which is converted into medermycin via *C*-glycosylation at C10; Route 2—-DHK is glycosylated firstly to form dihydro-medermycin, which is then lactonized into medermycin. Both route 1 and route 2 suggested that these two antibiotics (kalafungin and medermycin) share common earlier and middle stages (from the start units till DHK) in their biosynthetic pathways in AM-7161 (Fig. 7). Both two routes could explain the reason that the production of kalafungin was strictly controlled by the medermycin gene cluster in this strain.

Subsequently, we need to collect more evidences to investigate whether one route is preferred in AM-7161 or both of them process in the same way. Up to now, we have not detected the accumulation of dihydro-medermycin in AM-7161, probably implying the route 1 is preferred.

Efficient accumulation of kalafungin in AM-7161 suggested that *C*-glycosylation should be a rate-limiting step for medermycin production (Fig. 1 and Fig. 7), acting as a target for improvement of the production of medermycin and kalafungin using genetic approaches in the future.

In order to confirm the speculation in Fig. 7 and increase the yields of medermycin and kalafungin, it is necessary to perform functional characterization of several genes involved in *C*-glycosylation and investigate their expression levels both in AM-7161 and in the mutant M2.

In conclusion, we reported a medermycin- producing strain could accumulate kalafungin for the first time and obtained a kalafungin-high-yielding mutant derived from this strain. Significantly, we found that these two antibiotics were biosynthesized under the control of a common gene cluster, suggesting they share partially the same biosynthetic pathway in this strain. These results could inspire us to further reveal the biosynthetic mechanisms of these two bioactive compounds and improve their yields in the future.

## Supporting Information

S1 File Fig. A and Fig. B. (DOC)
