# Supplementary material for: Accumulation of a Bioactive Benzoisochromanequinone Compound Kalafungin by a Wild Type Antitumor-Medermycin-Producing Streptomycete Strain
Source: PLoS One. 2015 Feb 19;10(2):e0117690. doi: 10.1371/journal.pone.0117690 (PMC4335000; doi:10.1371/journal.pone.0117690)
Supplement: S1 File — (DOC) [file pone.0117690.s001.doc]

***Supplementary Data***

**Title**: Accumulation of a bioactive benzoisochromanequinone compound kalafungin by a wild type antitumor-medermycin-producing streptomycete strain

**Authors**: Jin Lü^a，b^, Qiang He^b^, Luyao Huang^b^, Xiaofeng Cai^b^, Wenwen Guo^b^, Jing He^c^, Lili Zhang^d^, Aiying Li^a, b,#^

**Affiliations**:

^a^State Key Laboratory for Microbial Technology, Shandong University-Helmholtz Joint Institute of Biotechnology and School of Life Sciences, Shandong University, Jinan 250100, China

^b^School of Life Sciences, Central China Normal University, Wuhan 430079, Hubei, China

^c^State Key Laboratory of Agricultural Microbiology, Huazhong Agricultural University, Wuhan 430070, Hubei, China

^d^Xinjiang Production & Construction Corps Key Laboratory of Protection and Utilization of Biological Resources in Tarim Basin, Tarim University, Alar 843300, Xinjiang, China

^#^Corresponding author: Aiying Li, Email: [ayli@sdu.edu.cn](mailto:ayli@sdu.edu.cn)
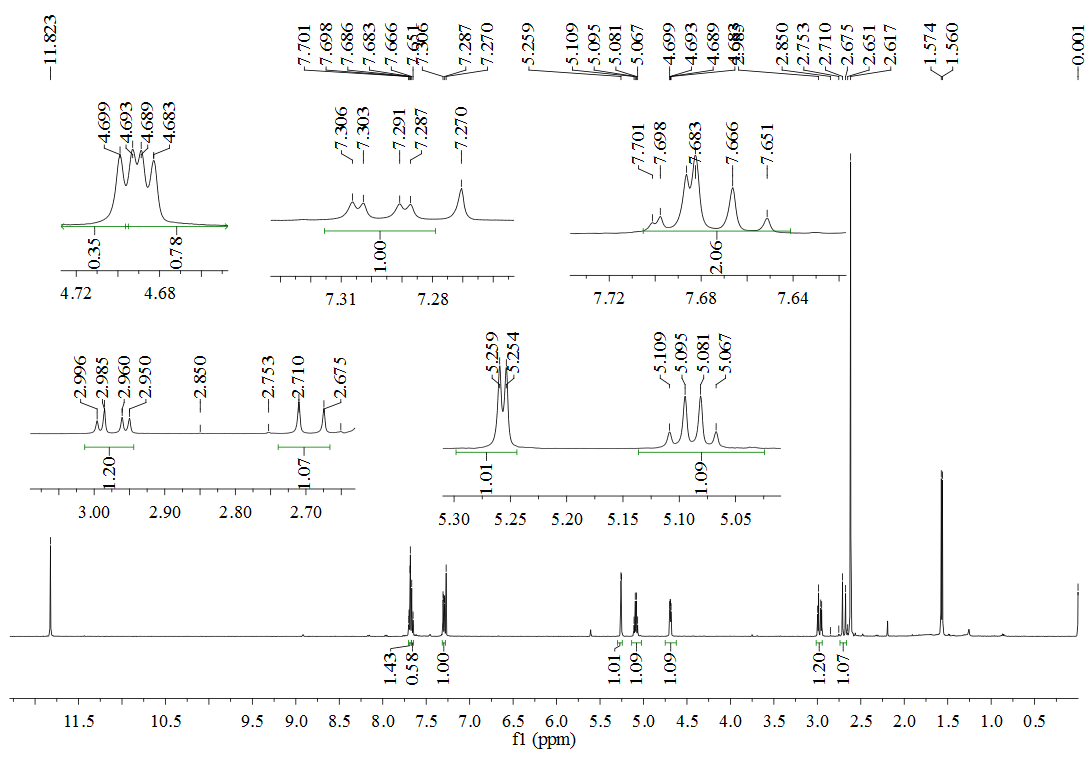


**Figure S1 ^1^H NMR spectrum of compound X (500 Hz, CDCl_3_).** Expected peaks for each hydrogen in compound X were detected. Two extra peaks (2.62 ppm and 7.27 ppm) were collected as well, representing chemical shifts of DMSO (residual solvent used during compound purification) and CDCl_3_ (deuterated NMR solvent) respectively.


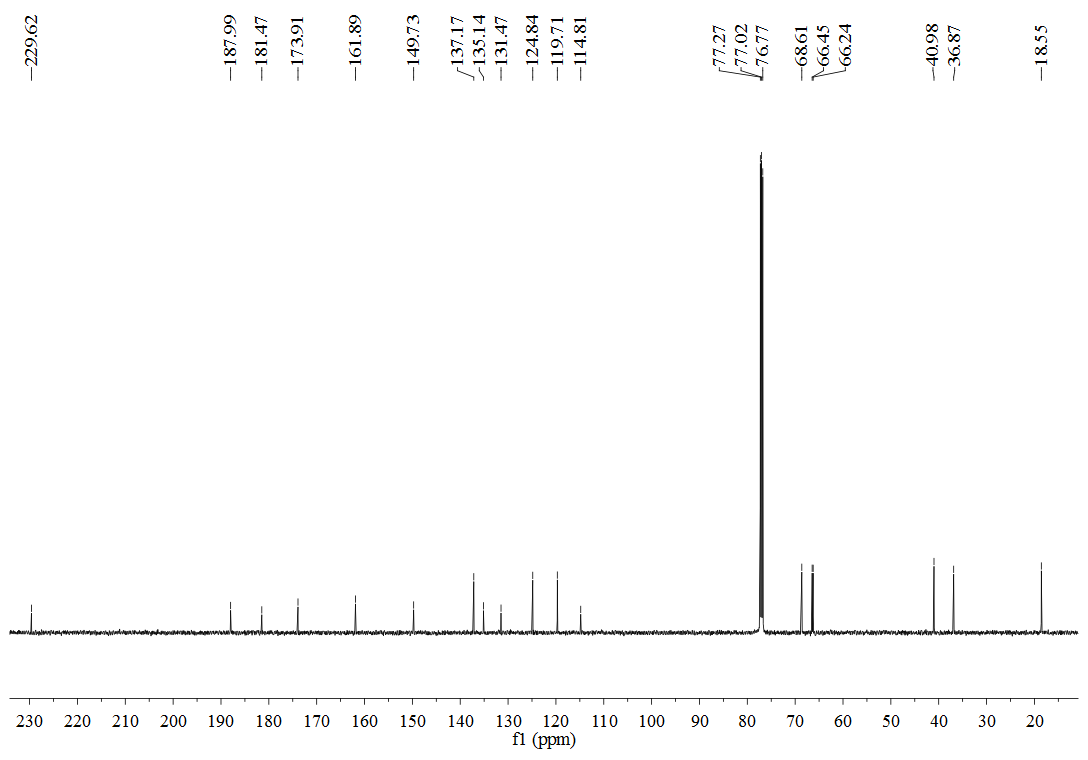


**Figure S2 ^13^C NMR spectrum of compound X** **(125 Hz, CDCl_3_).** Expected peaks representing chemical shift of each carbon in compound X were collected, besides two extra peaks (40.98 and 76.77 ppm for the residual solvent DMSO and deuterated solvent CDCl_3_ respectively).
